# Simultaneously Targeting Two Coupled Signalling Molecules in the Mesenchymal Stem Cell Support Efficiently Sensitises the Multiple Myeloma Cell Line H929 to Bortezomib

**DOI:** 10.3390/ijms24098157

**Published:** 2023-05-02

**Authors:** P. M. Rojas-Zambrano, J. E. Meyer-Herrera, P. F. Ruiz-Aparicio, J. P. Vernot

**Affiliations:** 1 Grupo de Investigación Fisiología Celular y Molecular, Facultad de Medicina, Universidad Nacional de Colombia, Bogotá 111321, Colombia; pmrojasz@unal.edu.co (P.M.R.-Z.); jemeyerh@unal.edu.co (J.E.M.-H.); pfruiza@unal.edu.co (P.F.R.-A.); 2 Instituto de Investigaciones Biomédicas, Facultad de Medicina, Universidad Nacional de Colombia, Bogotá 111321, Colombia

**Keywords:** mesenchymal stem/stromal cells, microenvironment, multiple myeloma, PKC, NF-κB, bortezomib, cell adhesion, drug susceptibility, HKPS, BAY11-7082

## Abstract

Several studies have shown that diverse components of the bone marrow (BM) microenvironment play a central role in the progression, pathophysiology, and drug resistance in multiple myeloma (MM). In particular, the dynamic interaction between BM mesenchymal stem cells (BM-MSC) and MM cells has shown great relevance. Here we showed that inhibiting both PKC and NF-κB signalling pathways in BM-MSC reduced cell survival in the MM cell line H929 and increased its susceptibility to the proteasome inhibitor bortezomib. PKC-mediated cell survival inhibition and bortezomib susceptibility induction were better performed by the chimeric peptide HKPS than by the classical enzastaurin inhibitor, probably due to its greatest ability to inhibit cell adhesion and its increased capability to counteract the NF-κB-related signalling molecules increased by the co-cultivation of BM-MSC with H929 cells. Thus, inhibiting two coupled signalling molecules in BM-MSC was more effective in blocking the supportive cues emerging from the mesenchymal stroma. Considering that H929 cells were also directly susceptible to PKC and NF-κB inhibition, we showed that treatment of co-cultures with the HKPS peptide and BAY11-7082, followed by bortezomib, increased H929 cell death. Therefore, targeting simultaneously connected signalling elements of BM-MSC responsible for MM cells support with compounds that also have anti-MM activity can be an improved treatment strategy.

## 1. Introduction

Multiple myeloma (MM) is a haematological malignancy of clonal plasma cells, an incurable disease whose aetiology is not completely known [1,2]. Much progress has recently been made in understanding MM with the characterization of the genetic abnormalities that lead to uncontrolled cell growth, which shows that in addition to being a clinically heterogeneous disease, it is also genetically highly diverse [3,4]. Interestingly, it has been signalled that targeting the biology of plasma cells in the bone marrow (BM) rather than its tumour genetic characteristics is more effective clinically [5]. Earlier work has shown that the survival of MM cells is dependent on the direct interactions of malignant plasma cells with the BM microenvironment (ME) and that, in turn, the BM-ME has a crucial role in pathogenesis [6,7,8,9]. This cell-to-cell interaction is facilitated by SDF-1/CXCR4 axis regulation of chemotaxis to BM [10] and by the high expression of MIF by MM cells, allowing both the interaction with ME stromal cells and the up-regulation of adhesion molecules on MM cells [11]. In experimental murine models of MM, it has been shown that CXCR4 inhibition by AMD3100 disrupts the interaction of MM cells with BM and enhances MM mobilization to circulation and sensitivity to therapeutic agents [12]. Several cell adhesion molecules (VLA-4 and VLA-5, for instance), soluble factors (such as IL-6) and extracellular matrix components (for example, fibronectin) have been shown to be relevant during BM-ME and MM cell interactions and responsible for MM cell survival and importantly, for the so-called cell adhesion-mediated drug resistance (CAM-DR) [13,14,15,16]. Intracellular signalling pathways (mainly STAT3, MAPK and PI3K) induced by these different echelons of interaction are crucial for the activation and enhancement of these properties [17,18,19]. The specific involvement of NF-κB [20], PKC [21], and the RACK1 chaperone of PKC and other protein kinases [22] was also demonstrated. This MM-induced signalling alters most ME resident cells, modifying their functionality and favouring the growth of malignant cells to the detriment of normal hematopoietic cells [8].

Although MM cells interact with many BM cells, it has been shown that BM mesenchymal stem/stromal cells (BM-MSC) have the greatest effect on MM cell growth, proliferation, and drug resistance, even changing their transcriptional profile and increasing their own cell proliferation [8,23,24,25,26]. Remarkably, these modifications emerging in BM-MSC persisted after patients’ treatment and even complete remission [27], suggesting the establishment of a long-lasting and permissive BM milieu suitable for relapse. The role of BM-MSC in MM progression is not only due to modifications in their expression profile or the secretion of soluble factors or vesicles but also to their capacity to recruit immune and other cells to the BM, modifying further immune functions and the BM-ME [27,28,29]. Additionally, it has been envisaged that the BM-ME influences drug resistance and is thus responsible for the recurrence/relapse of MM [30]. It is extremely important, in the search for novel strategies against this disease, to determine how drug resistance mechanisms are activated and what they are based on. Specifically, NF-κB has been shown to confer resistance to bortezomib (BTZ) through a BM-MSC-secreted IL-8-dependent stimulation pathway or via its activation by an autophagy-related mechanism [31,32]; this explains in part the effectiveness of NF-κB inhibitors in MM. However, some malignant cells may develop resistance to anti-NF-κB agents due to genetic modifications affecting different molecules along this pathway in MM cells [33,34], to the central role of NF-κB in increasing the expression of essential regulators of survival (anti-apoptotic molecules and DNA-repair proteins) [35], and also to the assisted supportive effect of the BM-ME, either by soluble factors, exosomes or direct cell contacts [36,37,38]. Therefore, targeting both the BM-ME and the MM cells is believed to be a more consistent strategy to eradicate MM cells [39]. In fact, thalidomide, BTZ, and lenalidomide, prototypic drugs approved for the treatment of this disease, target both MM cells and the BM-ME [9].

It has been shown that BTZ-resistant NF-κB activity is associated with cellular resistance to BTZ, suggesting that the identification of elements that activate NF-κB might have therapeutic value [31]. We hypothesize that since no compound can completely inhibit an intracellular signalling pathway, the use of other compounds acting either upstream or downstream in the same pathway may more efficiently inhibit BM-MSC support and BM-MSC-induced drug resistance. Since others and we have shown that BM-MSC PKC activity is partially responsible for the BM-MSC supportive role and drug resistance in various haematological malignancies [40,41], and because PKC activity is carried out upstream of NF-κB [42], here we have studied the effect of inhibiting these connected signalling molecules in BM-MSC on the sensitivity of MM cells to BTZ treatment.

## 2. Results

### 2.1. BM-MSC Favour Slightly the Growth of H929 Cells Greatly the Resistance to BTZ

**Figure 1:**
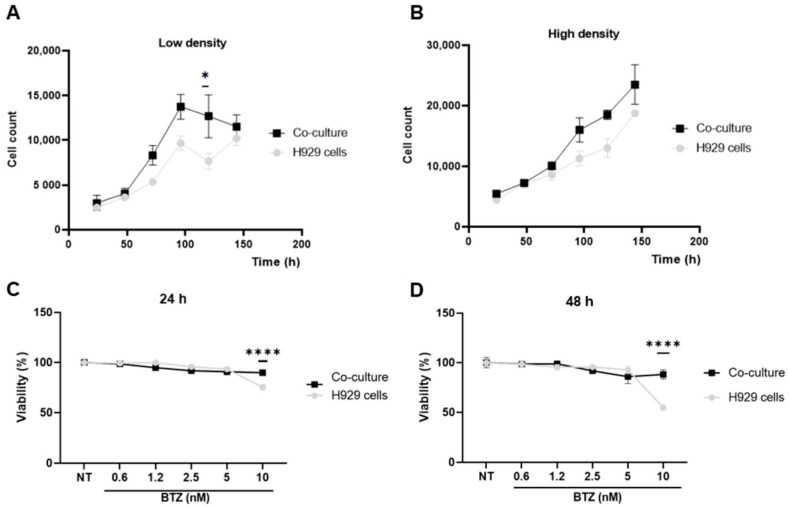
BM-MSC support growth and protect H929 cells from BTZ cytotoxicity. Co-cultures were established for 6 days by seeding H929 cells (**A**) at low density (2.000/100 µL) or (**B**) high density (4.000/100 µL) on a BM-MSC monolayer. H929 cells were harvested and counted in a Neubauer chamber using Trypan blue dye every 24 h, and the population doubling time was calculated. As a control, H929 cells were seeded in monoculture. BM-MSC and H929 co-cultures were established and treated with different concentrations of BTZ for (**C**) 24 h or (**D**) 48 h. H929 cell viability was determined by the MTT assay. H929 cells in monoculture and untreated co-cultures were used as controls. Data are expressed as the mean ± SEM of triplicates cultures. Statistical analysis was performed using a one-way ANOVA test followed by a Kruskal–Wallis test (**A**,**B**) *p*-value * < 0.05; and a two-way ANOVA (**C**,**D**) with Dunnett’s post hoc test. *p*-value **** < 0.0001.

We initially examined the effect of a co-culture system established with previously characterized phenotypically and functionally BM-MSC (Supplementary Figure S1A,B) on the growth of the MM cell line H929. In general, there were no statistically significant differences in the growth of H929 cells, although there was a tendency for H929 cells to grow slightly better in co-culture with BM-MSC, being significant at low densities and in a particular time-point (120 h; Figure 1A,B). We calculated a population doubling time of 61.7 ± 1.1 h for H929 cells alone and 58.1 ± 1.2 h for H929 cells in co-culture.

Then, we confirmed the susceptibility to BTZ of the H929 cell line and found that this was evident and strong at 10 nM BTZ or higher, with almost 90% of the cells losing viability after 72 h of treatment, compared to untreated cells (Supplementary Figure S2A). We found an IC_50_ of 6 nM (Supplementary Figure S2B), which is in agreement with previous reports [43,44]. On the contrary, the H929 cell line was not susceptible to DEXA, even at higher concentrations, with only a slight effect seen at 50 µM after a 72h treatment period (Supplementary Figure S2C). We next assessed if a co-culture with BM-MSC could protect H929 MM cells from BTZ treatment. Since a 72 h treatment period induced high mortality in the H929 cell line (>90%), we reduced the time of exposure to BTZ to 48 and 24 h. First, we showed that BM-MSC were not affected by BTZ treatment during the time periods tested of 24 or 48 h (Supplementary Figure S3A,B). Next, co-cultures were set at the appropriate cell densities and then treated with BTZ at different concentrations (0.6–10 nM; Figure 1C,D). As can be seen, no effect on H929 cell viability was observed at concentrations below or equal to 5 nM BTZ. The treatment of H929 cells with 10 nM BTZ for 24 h (Figure 1C) or 48 h (Figure 1D) induced 25% and 45% loss of cell viability, respectively. Importantly, in both cases, cell viability was fully preserved by co-culturing H929 cells with BM-MSC (Figure 1C,D).

### 2.2. BM-MSC Can Even Restore the Viability of H929 Cells Previously Treated with BTZ

**Figure 2:**
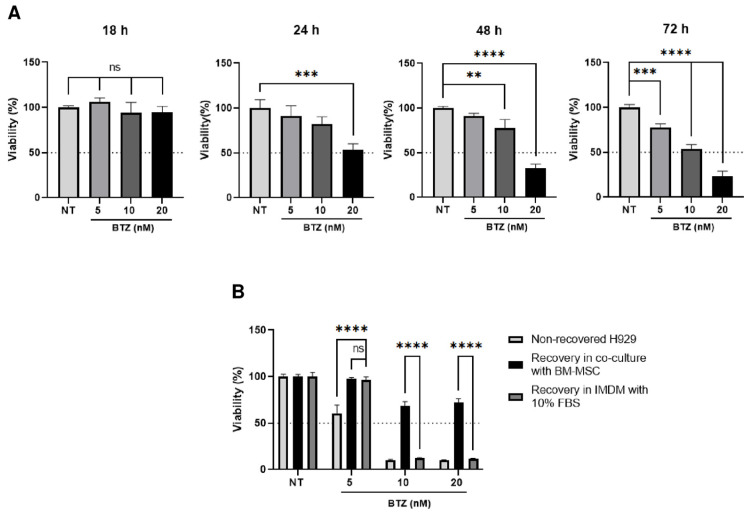
BM-MSC are able to recover H929 cell viability after strong BTZ treatment. (**A**) H929 cells were treated with different concentrations of BTZ for different periods of time, as indicated. Cell viability was assessed by the MTT assay. (**B**) H929 cells were pre-treated with different concentrations of BTZ for 72 h, washed and then co-cultured or not with BM-MSC for 24 h. Pre-treated H929 cells cultured in IMDM with 10% FBS for 24 h were used as controls. Data are expressed as the mean ± SEM of triplicate cultures. Statistical analysis was performed using a one-way ANOVA test followed by a Kruskal–Wallis test (**A**) and a two-way ANOVA followed by Dunnett’s post hoc test (**B**). No significant statistical differences (ns) and *p*-values ** < 0.01, *** < 0.001, **** < 0.0001.

We further explored if BM-MSC could restore the viability of MM H929 cells that had been previously treated with BTZ for periods of time, inducing high mortality. To do this, H929 cells were treated with different BTZ concentrations (5, 10 and 20 nM) and during different periods (18, 24, 48, 72 h). The idea was to find a BTZ treatment (time and concentration) in which a clear deleterious effect on H929 cell viability could be observed at all the conditions tested. This only occurred after a treatment with BTZ for 72 h (Figure 2A). Hence, H929 cells, which had been treated with BTZ for 72 h, were gently washed, resuspended in a complete culture medium and set back in culture for 24 h in the presence of BM-MSC (Figure 2B, middle columns). As a control, H929 cells were cultured only with a complete culture medium (Figure 2B, right columns). At the end of the incubation period with BTZ, viability was importantly reduced to 61% at 5 nM BTZ and to 10% at 10 and 20 nM BTZ (Figure 2B, left columns). As can be seen, BM-MSC were able to restore cell viability of H929 cells at 5 nM BTZ, but this effect was not different from the one observed only with complete medium (Figure 2B, 5 nM, middle and right columns). Nevertheless, at higher BTZ concentrations (10 and 20 nM), recovery was only observed in co-cultures with BM-MSC (Figure 2B, middle columns), showing the specific protective effect of the mesenchymal stromal support and dissociating the effect that could be produced by the culture medium and/or the FBS.

### 2.3. The BM-MSC Supportive Capacity and Resistance to BTZ Involves PKC Activity

To test if the supportive capacity given by BM-MSC was dependent on PKC activity, we inhibited this enzyme in BM-MSC with the conventional inhibitor enzastaurin (ENZA) [45] or with an inhibitory chimeric peptide (called HKPS), the latter being specific for the cPKC isoforms [46]. We have first shown that cell viability of BM-MSC was not affected by ENZA alone or ENZA plus BTZ treatment (Supplementary Figure S3C); we have previously shown that HKPS treatment for 2 h at 40 µM has a minimum deleterious effect on BM-MSC viability [40].

**Figure 3:**
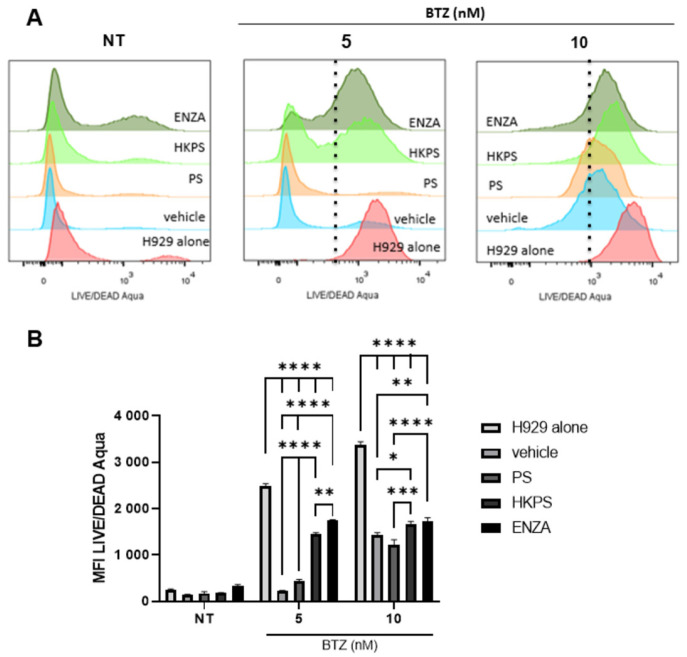
PKC inhibition of BM-MSC by ENZA or HKPS increases H929 cell sensitivity to BTZ in co-cultures. (**A**) BM-MSC were pre-treated or not with PKC inhibitors (HKPS or ENZA) before being co-cultured with H929 cells and then treated for 72 h with BTZ. H929 cells alone or in co-cultures with BM-MSC pre-treated with control PS peptide or vehicle (0.4% DMSO) were used as controls. H929 cell viability was assessed by flow cytometry using the LIVE/DEAD Aqua staining (**B**) Quantification of mean fluorescence intensity (MFI) of LIVE/DEAD Aqua positive cells in the different conditions described in (**A**). Data are expressed as mean ± SEM obtained from two independent experiments with three replicates (*p* values: nonparametric two-way ANOVA followed by Dunnett’s post hoc test. * < 0.05, ** < 0.01, *** < 0.001, **** < 0.0001).

To clearly differentiate the effect that these PKC inhibitors could have on H929 cells viability by inhibition of the BM-MSC PKC enzyme, these experiments were performed and analysed by flow cytometry after cell labelling with CD73 (a BM-MSC marker) and the reagent Aqua Fixable Live/Dead to clearly discriminate H929 cells. From this assessment, it was clear that the BM-MSC protective effect on H929 cells is lost after the pre-treatment of BM-MSC with both PKC inhibitors, with ENZA having a stronger effect than the HKPS peptide, with 2.5 times more affected cells after ENZA treatment (Figure 3A, NT panel; Supplementary Figure S4). The treatment with 5 nM BTZ affects plasma cell membrane permeability of almost all H929 cells cultured alone, but co-cultivation with control BM-MSC inhibited this effect (Figure 3A,B, vehicle and control PS). On the contrary, the co-cultures of H929 cells with BM-MSC that had been previously treated with ENZA or HKPS did not protect H929 cells from BTZ, with ENZA having a higher inhibitory effect at 5 nM BTZ. In particular, an H929 cell sub-population (about 40%), not affected by BM-MSC HKPS pre-treatment, was evident at 5 nM BTZ (Figure 3A, HKPS, left cell population). This cell sub-population was reduced four times in ENZA-pre-treated BM-MSC. The loss of the protective effect of ENZA or HKPS pre-treated BM-MSC could also be seen at 10 nM BTZ, although to a lower extent (Figure 3A,B).

### 2.4. BM-MSC PKC Mediates Cell-to-Cell Adhesion and BTZ Resistance of H929 Cells

**Figure 4:**
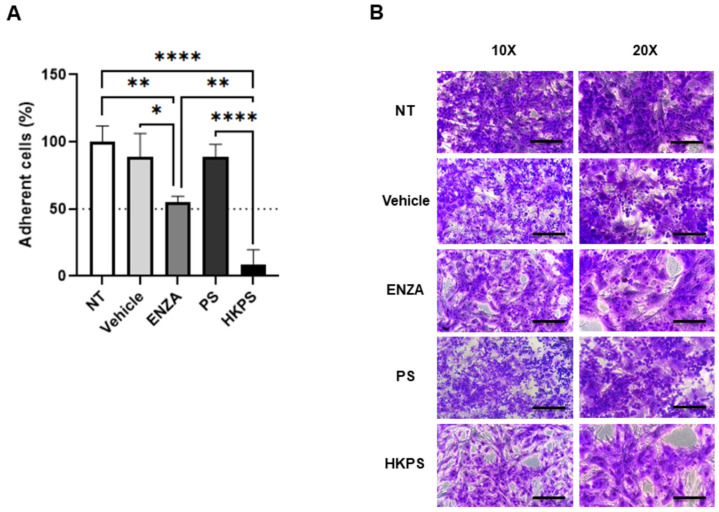
PKC inhibition in BM-MSC disrupts the adhesion of H929 cells. (**A**) BM-MSC were treated or not (NT) with 0.4% DMSO, 20 µM ENZA, 40 µM HKPS or 40 µM PS for 2 h, and then H929 cells were seeded over this 90% confluent monolayer and incubated for an additional 2 h period. Non-adherent H929 cells were recovered by washing the wells with PBS 1× and then counted using a haemocytometer. The percentage of adhesion was calculated based on the initial input of H929 cells and then normalized to NT cells. (**B**) The remaining adherent cells in the co-cultures were fixed and stained with crystal violet. Micrographs from a representative experiment are shown at 10× (100 µm scale bars) or 20× magnification (50 µm scale bars). Data are expressed as the mean ± SEM of three replicates. Statistical analysis was performed using a one-way ANOVA test followed by a Kruskal–Wallis test. *p*-values * < 0.05, ** < 0.01, **** < 0.0001.

Since it has been shown in several models of haematological malignancies that susceptibility to drug treatment is increased after detachment of malignant cells from stromal support [13], we have evaluated the role of the PKC inhibitors on H929 cell adhesion to BM-MSC. Indeed, both compounds strongly inhibited H929 cell adhesion to BM-MSC (46% for ENZA and 92% for HKPS; Figure 4A,B). Both vehicle and PS peptide controls had minimum effects on cell adhesion, as seen in the microphotographs (Figure 4A,B), in complete agreement with their ineffectiveness in inducing susceptibility to BTZ (Figure 3). All in all, these experiments suggest that resistance to BTZ treatment is dependent at least in part on the capacity of H929 cells to bind to the stromal cell support and that if adhesion is hindered by, for example, PKC inhibitors, then susceptibility to BTZ is increased.

### 2.5. Pre-Treatment of BM-MSC with BAY11-7082 also Inhibits Cell Adhesion and Increases BTZ Susceptibility of H929 Cells

**Figure 5:**
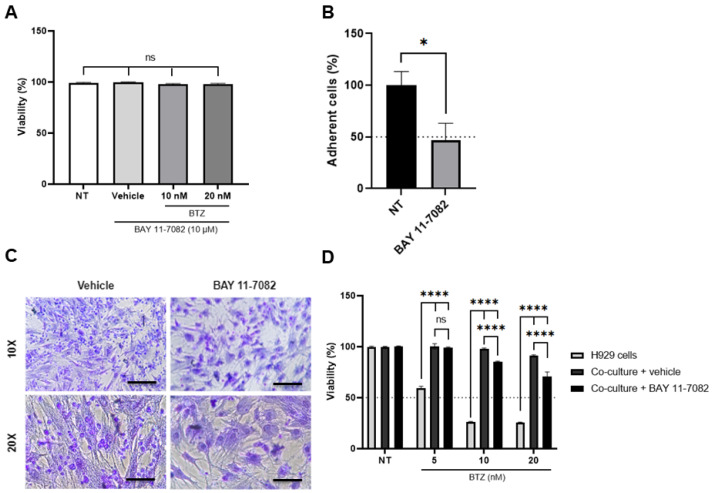
BM-MSC NF-kB inhibition with BAY11-7082 decreases the adhesion of H929 cells and their protective effect against BTZ. (**A**) BM-MSC were treated first with BAY11-7082 for 4 h and subsequently with (or not) 10 and 20 nM BTZ. As a control, BM-MSC were treated with the vehicle (0.02% DMSO). (**B**) BM-MSC were treated with BAY11-7082 for 4 h, and then co-cultures were established for an additional 2 h. Non-adherent H929 were recovered by washing the wells with PBS 1X and then counted using a haemocytometer. The percentage of adhesion was calculated based on the initial input of H929 cells, and data were normalized to vehicle-treated cells. (**C**) Adherent H929 cells in the co-cultures were fixed and stained with crystal violet. Representative microphotographs of one representative experiment are shown at 10× (100 µm scale bars) or 20x magnification (50 µm scale bars). (**D**) H929 cells were co-cultured with BM-MSC previously treated for 4 h with 10 µM BAY11-7082, and co-cultures were then treated with BTZ at the indicated concentrations for an additional 72 h. As controls, co-cultures established with BM-MSC pre-treated with the vehicle (0.02% DMSO), and H929 cells in monoculture were used. Data are expressed as the mean ± SEM of triplicate cultures. Statistical analysis was performed using a one-way ANOVA test followed by a Kruskal–Wallis test (**A**,**B**) and a two-way ANOVA followed by Dunnett’s post hoc test (**D**). No significant statistical differences (ns) and *p*-value * < 0.05; *p*-values **** < 0.0001.

Since NF-κB acts downstream of PKC and is also involved in the expression of adhesion molecules [47], we have also explored if this transcription factor could also be involved in the adhesion of H929 cells to BM-MSC. We directly inhibited the phosphorylation of IkB1 and IkB2 and, therefore, its proteasomal degradation with the BAY11-7082 compound. We have previously shown that the treatment of BM-MSC with 10 µM BAY11-7082 for 4 h totally abolished NF-κB activity [40]. First, we showed that BM-MSC viability was not affected by treatment with BAY11-7082 and/or BTZ at the concentrations and times employed in these experiments (Figure 5A). BAY11-7082 inhibited (near 52% inhibition) H929 cell adhesion to BM-MSC (Figure 5B,C). This inhibition of cell adhesion was similar to that induced by ENZA but less than that produced by the HKPS peptide (Figure 4).

Then, we studied if the BTZ susceptibility of H929 cells was affected by pre-treatment of BM-MSC with BAY11-7082. At 5 nM BTZ, >40% of H929 cells died in monoculture; co-cultivation with BM-MSC protected H929 cells from BTZ, and pre-treatment with BAY11-7082 did not affect this protective effect (Figure 5D). At higher 10 and 20 nM BTZ, high (75%) H929 cell death occurred, and co-cultivation with BM-MSC was able to protect H929 cells. Here, an important reduction in the protective effect of BM-MSC over H929 cells was observed when BM-MSC were previously treated with BAY11-7082 (Figure 5D). This loss of viability of H929 cells was about 15% and 30% for 10 and 20 nM BTZ, respectively, suggesting an involvement of the NF-κB pathway in the protective effect of BM-MSC at higher BTZ concentrations.

### 2.6. Pre-Treatments of BM-MSC with ENZA or HKPS Differentially Affect the Activation of NF-κB Signalling Pathway Induced in BM-MSC by Co-Cultivation with H929 Cells

**Figure 6:**
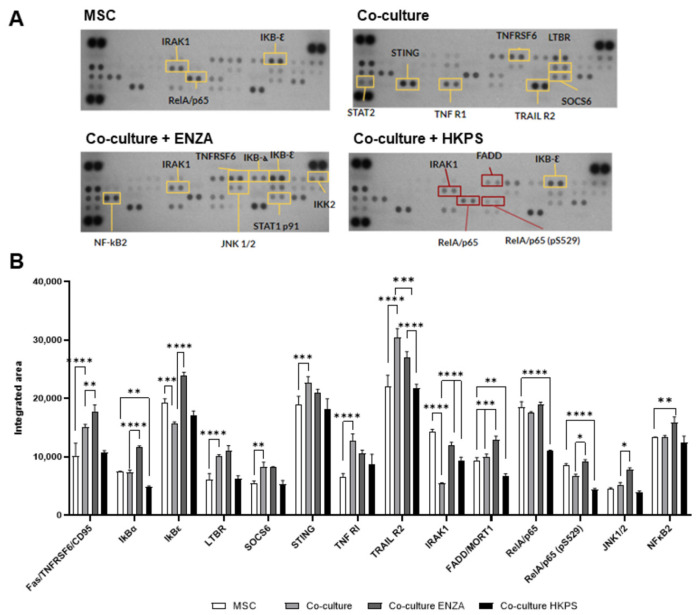
BM-MSC interaction with H929 cells induces changes in BM-MSC expression of NF-κB signalling pathway-related molecules. BM-MSC pre-treated or not with PKC inhibitors (HKPS or ENZA) for 2 h were co-cultured for 2 additional hours with H929 cells. Then, H929 cells were gently removed, and BM-MSC were trypsinized and lysed for protein extraction. (**A**) Protein extracts were incubated in the Proteome Profiler Human NF-κB microarray; higher (yellow squares) and lower (red squares) protein expressions are shown. (**B**) Changes in the expression of NF-κB signalling pathway-related molecules in BM-MSC by dot density quantification. The integrated area was made using ImageJ software, and data are expressed as mean ± SEM. A non-parametric two-way ANOVA followed by Dunnett’s post hoc test was performed for statistical analysis. *p*-values * < 0.05, ** < 0.01, *** < 0.001, **** < 0.0001.

We searched for the BM-MSC signalling molecules that could be involved in cell adhesion and eventually in the induction of susceptibility to BTZ. For this purpose, a microarray of signalling molecules related to the NF-κB pathway was employed (Figure 6A). First, we looked for the signalling molecules that were modified in BM-MSC by co-cultivation with H929 cells. Six molecules were increased; Fas/TNFRSF6/CD95, LTBR, SOCS6, STING, TNF R1, and TRAIL R2, and two molecules were downregulated (IRAK1 and IkBε; Figure 6B). The pre-treatment of BM-MSC with ENZA did not modify the expression of these molecules; it was increased in one case (Fas/TNFRSF6/CD95) and decreased in another case (TRAIL). On the contrary, the pre-treatment of BM-MSC with the HKPS chimeric peptide decreased the expression of all of these molecules to basal levels (Figure 6B). For IRAK1 and IkBε, the pre-treatment of BM-MSC with the PKC inhibitors reverted the reduction induced by the co-cultivation with H929 cells. From these experiments, it is clear that the NF-κB signalling molecules that were increased in BM-MSC by co-cultivation with H929 cells were reduced successfully and consistently by the pre-treatment with the HKPS peptide. Other NF-κB signalling molecules did not vary in BM-MSC upon co-culture (FADD/MORT1, IkBα, JNK1/2, NF-κB2, RelA/p65 and RelA/p65(pS529) (Figure 6B). Nevertheless, pre-treatment of BM-MSC with PKC inhibitors had different effects on them; in general, ENZA pre-treatment increased the expression of these molecules, while HKPS reduced their expression to basal levels or even further.

### 2.7. Targeting of BM-MSC Support Alone or the Co-Cultures with HKPS and BAY11-7082, Is More Effective Than Individual Treatments in Inducing H929 Susceptibility to BTZ

**Figure 7:**
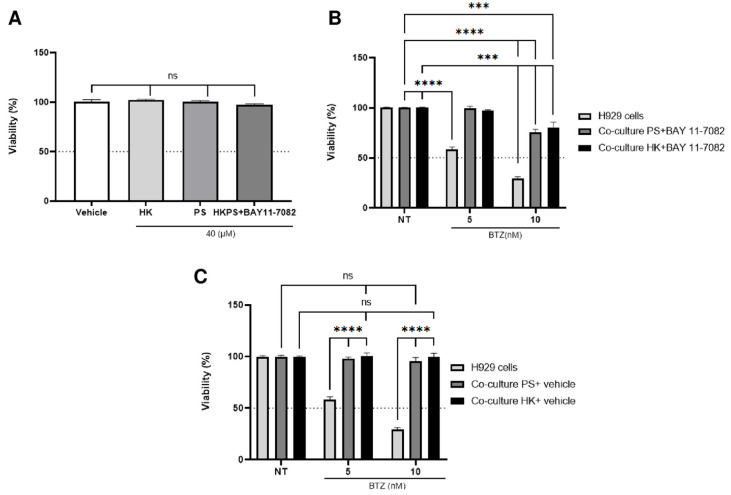
BAY11-0782 treatment of BM-MSC only slightly affects H929 cell sensitivity to BTZ. (**A**) BM-MSC alone were pre-treated with control peptides (HK or PS) or HKPS at 40 µM for 2 h, followed by treatment with BAY11-0782 (10 µM) or vehicle (0.02% DMSO) for an additional 4 h. (**B**) H929 cells were treated (or not) with BTZ for 72 h in the presence or absence of pre-treated BM-MSC with PS or HK for 2 h and then BAY11-0782 for 4 h or (**C**) without BAY11-0782. The MTT assay determined the viability. Data are expressed as the mean ± SEM of triplicate cultures. Statistical analysis was performed using a one-way ANOVA test followed by a Kruskal–Wallis test (A) and a two-way ANOVA followed by Dunnett’s post hoc test (**B**,**C**). No significant statistical differences (ns) and *p*-values *** < 0.001, **** < 0.0001.

From the above experiments, it is clear that although both PKC and NF-κB inhibitors have an important effect on H929 cells’ sensitization to BTZ, in part due to their capacity to inhibit cell adhesion, they seem to act at different BTZ concentrations. While HKPS has an important effect at low BTZ concentrations (<10 µM; Figure 3), BAY11-7082 has a main effect at concentrations above 10 µM (Figure 5D). Also, HKPS inhibits cell adhesion stronger and has an opposite effect in the regulation of signalling molecules of the NF-κB pathway, compared to ENZA. Therefore, it was mandatory to determine the joint effect of these two inhibitors (HKPS and BAY11-7082) on BM-MSC to induce H929 cells’ susceptibility to BTZ. First, we showed that BM-MSC did not lose their viability after treatment with both compounds (HKPS and BAY11-7082; Figure 7A). Also, BM-MSC treated with control peptides (HK and PS) and BAY11-7082 did not alter the viability of H929 cells to 5 nM BTZ but did it slightly (25%) at 10 nM BTZ (Figure 7B). Importantly, this effect did not occur when treatment with BAY11-7082 was omitted (Figure 7C). Suggesting that the effect produced in H929 cells, although weak, is due solely to BAY11-7082.

**Figure 8:**
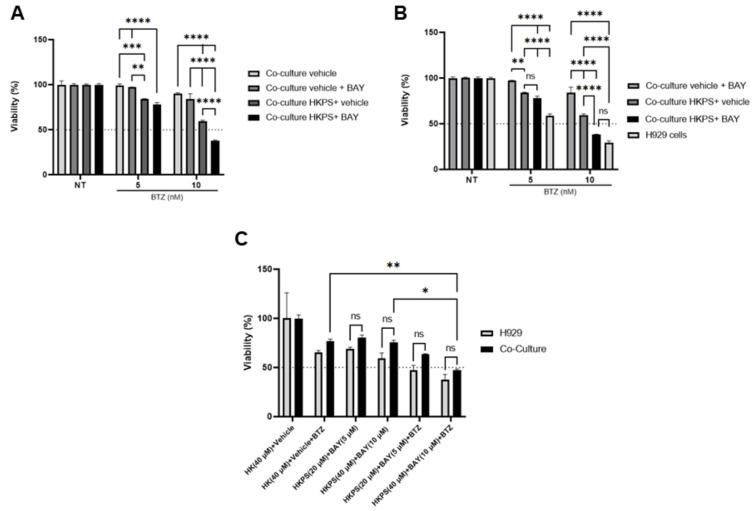
Treatment of BM-MSC or co-cultures with HKPS and BAY11-0782 increases H929 cell sensitivity to BTZ. (**A**) BM-MSC were treated or not (vehicle, 0.02% DMSO) with HKPS (40 µM) for 2 h, followed by treatment with BAY11-0782 (10 µM) or vehicle for an additional 4 h. Then, co-cultures were established and treated with BTZ for 72 h. (**B**) BTZ-treated co-cultures established with pre-treated BM-MSC, as indicated in the figure, were compared to H929 cells alone treated with BTZ. The MTT assay determined H929 cell viability. (**C**) Co-cultures were established with BM-MSC for 24 h before the treatment with HKPS (20 or 40 µM) and BAY11-7082 (5 or 10 µM) for 4 h. Then, inhibitors were removed, and co-cultures were treated or not with BTZ at 10 nM for an additional 24 h. H929 cell viability was determined by the MTT assay. Data are expressed as the mean ± SEM of triplicate cultures. Statistical analysis was performed using a two-way ANOVA followed by Dunnett’s post hoc test. No significant statistical differences (ns) and *p*-values * < 0.05, ** < 0.01 *** < 0.001, **** < 0.0001.

Subsequently, the effect of pre-treatment of BM-MSC with HKPS and/or BAY11-7082 on the susceptibility to BTZ of H929 cells was tested. Separate treatments showed a greater effect of HKPS (40%) than of BAY11-7082 (12%) at 10 nM BTZ (Figure 8A). Pre-treatment of BM-MSC with these two compounds was done by first incubating mesenchymal cells with HKPS for 2 h and then with BAY11-7082 for 4 h. This BM-MSC pre-treatment induced a higher susceptibility (70%) to 10 nM BTZ, showing an additive and eventually synergistic effect (Figure 8A). At 5 nM BTZ, the effect can also be seen, although to a lesser degree, and it seems to be only additive (Figure 8A). The susceptibility to 10 nM BTZ of H929 cells that had been cultured with BM-MSC treated first with HKPS and then with BAY11-7082 was equivalent to that of H929 cells cultured alone (Figure 8B), showing that the inhibition of both PKC and NF-κB in BM-MSC completely abrogates their protective effect over H929 cells.

The above results are even more relevant, taking into consideration that PKC inhibitors (HKPS and ENZA) have a direct effect on the growth of H929 cells at lower concentrations than those used here for inhibiting the BM-MSC support (Supplementary Figure S5A,B). Similarly, and as expected, the NF-κB inhibitor BAY11-7082 had an important inhibitory effect on the growth of this cell line, although in this case, we found an IC_50_ close to 50 µM (Supplementary Figure S5C). So, instead of using pre-treated BM-MSC, we tested the simultaneous use of the inhibitors HKPS and BAY11-7082 with the anti-MM compound BTZ in already established co-cultures of BM-MSC and H929 cells.

In the first set of experiments that we performed, it was impossible to get reliable information since although BM-MSC were not affected by the individual treatment of HKPS or BAY11-7082, or both, the concurrent addition of BTZ severely affected their viability, even when we used lower concentrations of HKPS (20 µM) and BAY11-7082 (5 µM; Supplementary Figure S6) and therefore the conclusions related to H929 cell viability under these circumstances were not adequately supported.

We then proceeded to do the next experiments by first treating the co-cultures with HKPS and BAY11-7082, washing out the inhibitors and then adding BTZ. With this procedure, BM-MSC viability was not affected. Importantly, we found significant differences between the co-cultures treated with HKPS and BAY11-7082 only and those treated additionally with BTZ; and between controls treatments (HK and vehicle) with BTZ and those treated with HKPS, BAY11-7082 and BTZ (Figure 8C). The optimal concentrations for HKPS and BAY11-7082 to achieve more H929 cell death were 40 µM and 10 µM, respectively. Importantly, there were no statistically significant differences between H929 cells alone treated with the three compounds and the co-cultures treated first with 40 µM HKPS and 10 µM BAY11-7082 and then with 10 nM BTZ (Figure 8C, last two columns to the right), showing again that the inhibition of BM-MSC support completely abrogates their protective effect and considerably increases the susceptibility to BTZ of H929 cells.

The results of Figure 8C,B are equivalent, but it must be taken into account that the former were obtained after a treatment period of 24 h, while the latter after one of 72 h.This indicates that in a quite more normal situation (BM-MSC and H929 cells interacting in the BM), the use of both inhibitors first (HKPS and BAY11-7082) followed by the drug BTZ could have an enhanced inhibitory effect on H929 cells growth (Figure 8C). The above results showed that treatments aiming to block several molecules of the same signalling pathway or molecules of different pathways that crosstalk at different levels might be more effective than blocking a single molecule of a specific signalling pathway. Additionally, these results showed the feasibility and effectiveness of targeting both the stromal support and the malignant cells with the same therapeutic tools.

## 3. Discussion

Understanding the molecular mechanisms of MM drug resistance is fundamental for the development of new strategies to treat this disease. One of the most crucial events in the development and progression of MM is the interaction of MM cells with the BM-ME [48]. For this reason, in addition to the different MM cell-intrinsic mechanisms of drug resistance [49], those based on the communication between BM-MSC and MM cells are gaining much more attention [50]. Adhesion molecules, tunnelling nanotubes, extracellular vesicles, exosomes, soluble factors and extracellular matrix components, among others, are facilitators of the communication between BM-ME and MM cells, with a significant impact on CAM-DR [14,49].

Here, we studied the supportive and drug-resistance roles of BM-MSC after the interaction with a MM cell line, showing that malignant H929 cells are efficiently protected from BTZ treatment. The protection given by BM-MSC was so robust that co-cultures were even able to rescue H929 cell viability after having undergone an intensive 72 h BTZ treatment. It has been proposed that deciphering the mechanisms of protection may be one key element to coping with malignant cell heterogeneity in a general way, taking into account that these mechanisms could be independent of the genetic defects of the malignant cells [51]. This could eventually be a more practical strategy than those aiming to develop individualized therapies, totally dependent on the particularities of the genetic defect and on the characteristics and biomarkers of the malignant cell [52]. To a large extent, we found that this shielding effect was based mainly on the adhesion capacity of H929 cells to BM-MSC since compounds that abrogate this cell-to-cell interaction were very effective in increasing BTZ susceptibility. In particular, we found that the pre-treatment of BM-MSC with the classical PKC inhibitor ENZA or a novel chimeric peptide HKPS inhibited cell adhesion and induced BTZ susceptibility. This is similar to what has been shown in diverse B cell malignancies [40,41], showing that CAM-DR can be overcome by inhibiting the interaction or cell communication between stromal and malignant cells by different means.

Moreover, we also showed that pre-treatment of BM-MSC with BAY11-7082, an IkBα phosphorylation and NF-κB inhibitor, also hindered adhesion between BM-MSC and H929 cells inducing H929 cells’ susceptibility to BTZ. Therefore, by inhibiting either one of these two molecules in BM-MSC, i.e., cPKC and NF-κB, it was possible to increase H929 cells’ susceptibility to BTZ. Although these molecules may act in the same cell-signalling pathway, with PKC acting upstream of NF-κB, they interact and are regulated by other signalling molecules [47,53], making it possible that their combined inhibition may have an additive effect when inducing BTZ susceptibility in H929 cells. To test this, we chose the HKPS peptide over ENZA for several reasons: first, HKPS had a stronger inhibitory effect on cell adhesion of H929 cells to BM-MSC; second, HKPS counteracts better the changes in expression of NF-κB signalling molecules induced by co-culturing BM-MSC and H929 cells; and third, since HKPS has been engineered with a natural PKC regulatory sequence [46], it is more specific for PKC, especially cPKC, than ENZA. Initially, the separated treatment with these compounds showed a greater effect of HKPS over BAY11-7082 in the induction of BTZ susceptibility, while the combined treatment showed an additive or even synergistic effect at particular BTZ concentrations, suggesting that these compounds may also affect other signalling molecules or pathways. The BTZ susceptibility of H929 cells co-cultured with pre-treated BM-MSC was equivalent to that of H929 cells cultured alone, indicating that the double treatment with HKPS and BAY11-7082 totally abrogates BM-MSC protection. Recently, a similar strategy targeting the BM-ME and affecting cell adhesion and MM proliferation by disrupting BM-MSC histone deacetylase 3 signalling also showed successful results [54].

Dual targeting approaches of BM-MSC and malignant cells have also shown effectiveness in leukemic models [40,41,51,55]. Early work has shown that the PKC inhibitor ENZA could have a direct effect on MM cells [56], either by preventing the phosphorylation of β-catenin and its degradation in the proteasome and, therefore, its accumulation, or by inhibiting Akt and inducing apoptosis [57,58]. Here we have shown that HKPS also induced cytotoxicity in the H929 cell line, with an IC_50_ of 17 µM, a concentration lower than that needed for affecting BM-MSC support. Therefore, considering that (1) HKPS, ENZA and BAY11-7082 were also directly cytotoxic for the H929 cells; (2) mutations in NF-κB signalling pathway are very common in MM [33,34]; (3) in many cases, drug resistance involves the participation of the canonical and non-canonical NF-kB signalling pathways in the malignant cells [59]; (4) neutralization of NF-κB and related signalling networks is important, because most of the growth factors and cytokines (APRIL, BAFF, IL6 and others) stimulating MM cell growth and long-term survival are dependent on the activation of this transcription factor [60,61]; (5) BM-MSC-mediated induction of NF-κB is important even for those MM cells harbouring mutational activation of this pathway [59,62]; and (6) positive feedback loops of stimulation are established by BM-MSC-derived soluble secreted factors and activation of NF-kB signalling in MM cells and stromal cells, we also performed experiments using the three compounds (HKPS, BAY11-7082 and BTZ) simultaneously in co-cultures, i.e., without pre-treatment of BM-MSC. Unfortunately, although BM-MSC were resistant to individual or double treatments with these compounds, the three inhibitors together compromised severely the viability of BM-MSC, impeding conclusions related to H929 cells’ susceptibility to BTZ. We do not know the reason for that, but it is probably due to some type of unwanted interactions between compounds or their particular effect on BM-MSC. Therefore, new experiments using these three compounds in the co-cultures were designed, first treating simultaneously the co-cultures with HKPS and BAY11-7082, washing them out, and then adding BTZ. These results were equivalent to the experiments in which BM-MSC were first pre-treated with the PKC and NF-κB inhibitors, suggesting that in a more real and physiological context, the treatment with these compounds is also effective in inducing susceptibility to BTZ, and they did so, by synergistically inhibiting the supportive signals given by BM-MSC and by directly having a cytotoxic effect on MM cells. This approximation is valuable, since drug resistance is resultant of multiple factors, some of them intrinsic to the malignant cells and others dependent on the BM-ME [35].

We have shown that NF-κB-dependent signalling molecules (Fas/TNFRSF6/CD95, LTBR, STING, TNF R1, STAT2 and TRAIL R2/D5) are upregulated by the interaction of BM-MSC with H929 cells, and notably, that HKPS is able to reduce this increased expression to basal levels, showing that upstream PKC signals are important for NF-κB activity, and suggesting that inhibition of signalling molecules that are coupled in the same signalling pathway may help to reduce toxicity and develop more tolerated therapies [48]. Additionally, we found that HKPS inhibition of PKC in BM-MSC is superior to BAY11-7082 inhibition of NF-κB in inducing BTZ susceptibility, suggesting that additional mechanisms of inhibition (different from the NF-κB pathway) should be operating with HKPS. In this sense, it will be interesting to decipher which additional pathways or signalling nodes involving PKC participate in BTZ susceptibility induction in BM-MSC.

Finally, it must be mentioned that normal plasma cells are also dependent on similar BM-MSC cues, and therefore these treatments could also affect them. Nevertheless, we have shown before that for some types of leukaemia, malignant cells are more dependent on these signals than normal cells [40]. This could be related to some particular biological features of malignant cells, as it happens with the difference in susceptibility to BTZ between normal and MM plasma cells, the latter subjected to intense endoplasmic reticulum stress due to high protein load and secretion [63,64]. We have shown here that BM-MSC are not affected by the diverse treatments employed and that particular managements could be envisaged in order to preserve their function, which is required for normal hematopoietic physiology. Therefore, targeting specifically the BM-MSC compartment responsible for MM cell support and drug resistance, with compounds acting through connected intracellular signalling pathways and also as anti-MM agents, can be a better treatment strategy.

## 4. Materials and Methods

### 4.1. BM-MSC Isolation and Characterization

BM-MSC were obtained from BM samples of healthy paediatric patients with bone lesions after obtaining their parents’ signed informed consent. The protocols for isolation and characterization of BM-MSC have been approved by the Ethics Committee, Faculty of Medicine, Universidad Nacional de Colombia. Briefly, mononuclear cells (MNC) were collected by Ficoll Hypaque density gradient centrifugation (Histopaque, d = 1.077 g/mL, Sigma-Aldrich, St. Louis, MO, USA), and MNC were seeded in 75 cm^2^ culture flasks in Iscove’s Modified Dulbecco’s Medium, IMDM (GIBCO, Thermo Fisher Scientific, New York, NY, USA) supplemented with 10% foetal bovine serum (FBS, GIBCO-Life Technologies, New York, NY, USA), non-essential amino acids and 1% sodium pyruvate (LONZA, Walkersville, MD, USA), and incubated for 48 h under standard conditions (37 °C and 5% CO_2_).

Adherent and confluent BM-MSC were trypsinized (Sigma-Aldrich, St. Louis, MO, USA) for their immunophenotypic characterization by flow cytometry using the following antibodies against cell surface markers: CD105 (APC mouse anti-human CD105; clone SN6, Invitrogen, Frederick, MD, USA), CD90 (FITC mouse anti-human CD90; clone F15-42-1, Abcam, Cambridge, MA, USA), CD73 (FITC mouse anti-human CD73; clone AD2, BD Pharmingen, San Jose, CA, USA), CD44 (FITC anti-human CD44; clone MEM-85, Invitrogen, Frederick, MD, USA), CD34 (APC mouse anti-human CD34; clone 581, BD Pharmingen, San Jose, CA, USA) and CD45 (PerCP mouse anti-human CD45; clone 2D1, BD Biosciences, San Jose, CA, USA). Flow cytometry was performed in FACSAria III-up equipment (BD Biosciences, San Jose, CA, USA), and the FACSDiva-FlowJo software version 10.8.1 (BD Biosciences, Sunnyvale, CA, USA) was used for data analysis.

Multipotent differentiation capacity was evaluated using osteogenic, adipogenic and chondrogenic induction media from a STEMPRO differentiation kit (Thermo Fisher Scientific Inc, GIBCO by Life Technologies, Waltham, MA, USA) as previously described [40]. For the different experiments, BM-MSC were used in early passages (2–4) to avoid replicative senescence.

### 4.2. Multiple Myeloma Cell Line (H929)

The human MM cell line H929 (ATCC CRL-9068, American Tissue Culture Collection, Rockville, MD, USA) was cultured and maintained in RPMI 1640 medium (GIBCO-Life Technologies, Grand Island, NY, USA), supplemented with 10% FBS and 0.05 mM β-mercaptoethanol at 37 °C and 5% CO_2_. Aliquots of the same batch were thawed periodically (every 2 months).

### 4.3. Establishment of the Co-Cultures of BM-MSC with H929 Cells

1 or 5 × 10^4^ BM-MSC/well were seeded in complete IMDM medium in 96 or 24 well plates, respectively (SPL Life Sciences Co., Gyeonggi-do, Korea) depending on the experiment and incubated for 24 h in standard conditions to let them adhere. For the establishment of the co-cultures, the medium was removed, and wells were washed once with PBS 1X. Then, H929 cells in RPMI-1640 medium supplemented with 2% or 10% FBS (depending on the experiment) were added to the monolayer of BM-MSC in a 1:5 ratio (BM-MSC: H929 cells) and incubated for 24–72 h at 37 °C and 5% CO_2_. Once the incubation time was completed, different evaluations were performed. As controls, H929 cells or BM-MSC alone were seeded in incomplete RPMI 1640 or IMDM medium.

### 4.4. Peptide Synthesis

Peptides were synthesized using the SPPS-Fmoc/tBu methodology [65]. Three peptides were used: The PKC regulatory pseudosubstrate sequence (PS) RKGALRQY, present in all classical-PKC (cPKC) isoforms, i.e., PKCα, PKCβ-I, PKCβ-II and γ; the hydrophobic signal sequence derived from the fibroblast growth factor from Kaposi’s sarcoma, (HK) AAVALLPAVLLALLAP and the chimeric peptide (HKPS) containing both previous sequences [40,46]. Peptides were purified (>90%) and characterized by RP-HPLC and MALDI-TOF mass spectrometry, respectively.

### 4.5. BM-MSC Pre-Treatment with PKC and NF-κB Inhibitors

1 × 10^4^ BM-MSC were seeded in complete IMDM medium for 24 h, washed once with PBS 1X and pre-treated either with 20 µM Enzastaurin (ENZA; Sigma-Aldrich, St. Louis, MO, USA) or 40 µM HKPS for 2 h or with the 10 µM NF-κB inhibitor BAY11-7082 (Abcam, Cambridge, UK) for 4 h under standard conditions. In some experiments, the cells were treated sequentially with the HKPS peptide for 2 h and then with BAY11-7082 for an additional 4 h. Vehicle (0.4% or 0.02% DMSO, depending on the experiment) and the HK or PS peptides (40 µM) were used as controls.

### 4.6. Population Doubling Time of H929 Cells

H929 cells were seeded in 96 well plates in monoculture or in co-culture with BM-MSC at a cell density of 2.000 or 4.000 cells/well in a complete RPMI 1640 medium. In co-cultures, H929 cells were harvested by repetitive washes and counting, and staining was performed only with H929 cells in a Neubauer chamber using Trypan blue dye at 24, 48, 72, 96, 120, and 144 h of incubation. Calculation of population doubling time was made using the formula:

$$PDT=\frac{t}{PD}$$

where *t* is the culture time in hours, and *PD* is population doubling, which was determined by:

$$PD=\frac{log\;Nf-log\;Ni}{log2}$$

*Nf* is the final cell number, and *Ni* is the number of cells seeded in culture after each incubation time.

### 4.7. Cytotoxicity Assays

Cell viability of H929 cells and BM-MSC alone or in co-culture was analysed after treatment with BTZ and/or the PKC or NF-κB inhibitors or controls during different periods of time using the MTT assay kit (Thermo Fisher Scientific, Portland, OR, USA), according to the manufacturer’s protocol. To estimate the individual effect of each drug on the viability of H929 cells alone, 5 × 10^4^ H929 cells were seeded in 96-well plates and treated with BTZ (0.6–20 nM), dexamethasone (DEXA, Sigma-Aldrich, St. Louis, MO, USA; 0.09–50 µM), ENZA (0.3–40 µM), HKPS (2.5–80 µM) and/or BAY11-7082 (5–160 µM) during 2 to 72 h depending on the drug and the experiment. After the incubation time, the MTT reagent (5 mg/mL) was added to the culture medium and plates were incubated for 4 h at 37 °C, 5% CO_2_. Plates were centrifuged at 1.000× *g* for 10 min, the supernatant was discarded, and Formazan crystals were solubilized with 100 µL of DMSO 100%. Absorbance was measured using a spectrophotometer (Ultramark, Bio-Rad, Hercules, CA, USA) at 550 nm, and cell viability was calculated as a percentage of non-treated (BTZ) or DMSO-treated cells (DEXA, ENZA, BAY11-7082). Based on the MTT assay results, the IC_50_ value was calculated by performing a logarithmic transformation of the concentration values, and absorbance data were normalized, assigning 0 to the lowest absorbance value from each data set with GraphPad Prism 9.0 version software.

To assess the effect of PKC and NF-κB inhibition on the protective role of BM-MSC to H929 cells under treatment with BTZ, H929 cells were co-cultured with BM-MSC pre-treated with inhibitors or controls, and then 5–20 nM BTZ was added and incubated for 24, 48, and 72 h. Additionally, H929 cell viability recovery was evaluated by culturing H929 cells pre-treated or not with BTZ for 72 h with either BM-MSC or IMDM with 10% FBS for an additional 24 h. After incubation periods, the MTT assay was performed. BTZ non-treated cells were used as controls.

We also evaluated the joint effect of inhibiting both PKC and NF-κB on the viability of H929 and BM-MSC co-cultures. Briefly, co-cultures were established for 24 h, then HKPS (20 or 40 µM), BAY11-7082 (5 or 10 µM), and 10 nM BTZ were added and incubated simultaneously (the 3 compounds all at once for 24 h) or sequentially (HKPS for 2 h and then BAY11-7082 for 4 h, removal by washing out, and the BTZ treatment for 24 h). Next, the MTT assay was performed, as described. As controls, cells treated with 40 µM HK and 0.02% DMSO in combination without BTZ were used.

In some experiments, cell viability was also evaluated with the LIVE/DEAD Fixable Aqua Dead Cell Stain (Molecular Probes, Eugene, OR, USA) by flow cytometry. After treatment with cytotoxic drugs as described above, cells from co-cultures were labelled with LIVE/DEAD Aqua stain, as well as with PE mouse anti-human CD73 (BD Pharmingen, San Jose, CA, USA) to differentiate BM-MSC from H929 cells. Co-cultures with non-treated BM-MSC were used as controls.

### 4.8. H929 Cell Adhesion Assay

Co-cultures were established with BM-MSC pre-treated or not with PKC inhibitors or BAY11-7082, as previously described. After 2 h of incubation, non-adherent H929 cells were recovered by collecting the cell suspension and further washing each well twice with cold PBS 1×. Then, recovered cells were counted with Trypan blue in a Neubauer chamber. The percentage of adhesion was calculated based on the H929 cells input. The remaining cells in the co-cultures were fixed with methanol and stained with 0.5% crystal violet for 15 min, after which wells were washed 3 times with 1× PBS and air-dried for 2 h before photographing them under a microscope.

### 4.9. Assessment of NF-κB Pathway Activation in BM-MSC

Activation of the NF-κB pathway was also evaluated in BM-MSC from co-cultures with H929 cells by using the Proteome Profiler Human NF-κB Pathway Array (ARY029, R&D Systems, Minneapolis, MN, USA) following the instructions of the manufacturer. 1 × 10^6^ BM-MSC were seeded in 75 cm^2^ culture flasks and incubated for 24 h in complete IMDM medium, treated with 20 and 40 µM of ENZA or HKPS for 2 h, and then co-cultured with H929 cells for 2 h. Then, H929 cells were removed by several washes with PBS 1X + EDTA, and BM-MSC were trypsinized for protein extraction and quantitation using the BCA method (Thermo Fisher Scientific, Waltham, MA, USA). Array membranes were incubated with 350 µg of BM-MSC protein overnight at 4 °C; the detection antibodies were added, and membranes were exposed to photographic film for 10 min. The photographic records were made with the Gene Tool 4.03.02.0 software (Syngene, Frederick, MD, USA) in a Gel Imaging System (GeneGenius, Syngene, CA, USA), and the integrated area was analysed with the ImageJ software (National Institutes of Health, Bethesda, MD, USA).

The role of the NF-κB signalling pathway on BM-MSC support was also evaluated by co-culturing H929 cells with BM-MSC pre-treated or not with 10 µM NF-κB inhibitor BAY11-7082 for 4 h (Abcam, Cambridge, UK). Then, the co-cultures were treated with 5, 10, and 20 nM of BTZ for 72 h in standard conditions. After the incubation time, the viability of H929 cells was determined by the MTT assay, as described before. H929 cells in monoculture and in co-culture with non-treated BM-MSC were used as controls.

### 4.10. Statistical Analysis

Statistical analyses and graphs were made using the GraphPad Prism 9.0 version software, and results were expressed as the mean ± standard error of the mean (SEM). For the population doubling time, viability and adhesion experiments, data were analysed using a non-parametric 1-way analysis of variance (ANOVA) with a Kruskal–Wallis test. Comparisons between grouped conditions in cytotoxicity assays and NF-κB pathway microarrays were done using a 2-way ANOVA test followed by Dunnett’s post hoc test. The integrated area for each molecule on the human NF-κB pathway array was determined with ImageJ software (1.53r version) protein Array Analyzer (V.1.1.c). The statistical significance was generated using GraphPad Prism 9.0 version software, *p* = 0.0001 (****), *p* = 0.001 (***), *p* = 0.01 (**) and *p* = 0.05 (*).

## 5. Conclusions

The inhibition of either cPKC (by chimeric HKPS or ENZA) or NF-κB (by BAY11-7082) in BM-MSC reduced cell survival and increased the H929 cell lines’ susceptibility to BTZ. Compared to the classical PKC inhibitor ENZA, the chimeric peptide HKPS had the greatest ability to inhibit cell adhesion and counteract the increased expression of NF-κB signalling molecules induced in BM-MSC by co-cultivation with H929 cells. Also, we found a differential effect of HKPS and BAY11-7082 on BTZ susceptibility induction, with HKPS having effects at low (<10 µM) and BAY11-7082 at high (>10 µM) BTZ concentrations. Importantly, the simultaneous inhibition of cPKC and NF-κB in BM-MSC had an additive or even synergistic effect in BTZ susceptibility induction. This was even more relevant when considering that H929 cells were also directly susceptible to the inhibition by HKPS and BAY11-7082. Therefore, targeting specifically the BM-MSC compartment responsible for MM cell support and drug resistance, with compounds acting through connected signalling pathways and also as anti-MM agents, could be a better treatment strategy against MM.

## Data Availability

Research data are available upon request to the authors.

## Supplementary Materials

The following supporting information can be downloaded at: https://www.mdpi.com/article/10.3390/ijms24098157/s1.

## Abbreviations

MM	Multiple myeloma
BM	Bone marrow
MSC	Mesenchymal stem cells
ME	Microenvironment
SDF-1	Stem-derived factor 1
CXCR4	Chemokine receptor 4
MIF	Macrophage migration inhibitory factor
VLA	Very late antigen
IL	Interleukin
CAM-DR	Cell adhesion-mediated drug resistance
STAT	Signal transducer and activator of transcription
MAPK	Mitogen-activated protein kinase
PI3K	Phosphoinositol-3-kinase
NF- κB	Nuclear factor kappa-light-chain-enhancer of activated B cells
(c)PKC	(Classical) protein kinase C
RACK1	Receptor for activated C kinase 1
BTZ	Bortezomib
DEXA	Dexamethasone
FBS	Foetal bovine serum
ENZA	Enzastaurin
DMSO	Dimethyl sulfoxide
NT	Non-treated
TNFR(SF6)	Tumour necrosis factor receptor (superfamily member 6)
LTBR	Lymphotoxin beta receptor
SOCS6	Suppressor of cytokine signalling 6
STING	Simulator of interferon genes
TRAIL(R)	TNF-related apoptosis-inducing ligand (receptor)
IRAK	Interleukin receptor-associated kinase
IκB	Inhibitor of kappa B
JNK	Janus kinase
APRIL	A proliferation-inducing ligand
BAFF	B-cell activating factor
MNC	Mononuclear cells
BCA	Bicinchoninic acid
