# Supplementary material for: Simultaneously Targeting Two Coupled Signalling Molecules in the Mesenchymal Stem Cell Support Efficiently Sensitises the Multiple Myeloma Cell Line H929 to Bortezomib
Source: Int J Mol Sci. 2023 May 2;24(9):8157. doi: 10.3390/ijms24098157 (PMC10178910; doi:10.3390/ijms24098157)
Supplement: Supplementary file 1 [file ijms-24-08157-s001.zip › Last version ijms-2307614-supplementary.pdf]

Simultaneously targeting of two coupled signalling molecules in the mesenchymal stem cell support efficiently sensitises the multiple myeloma cell line H929 to bortezomib

SUPPLEMENTARY FIGURES

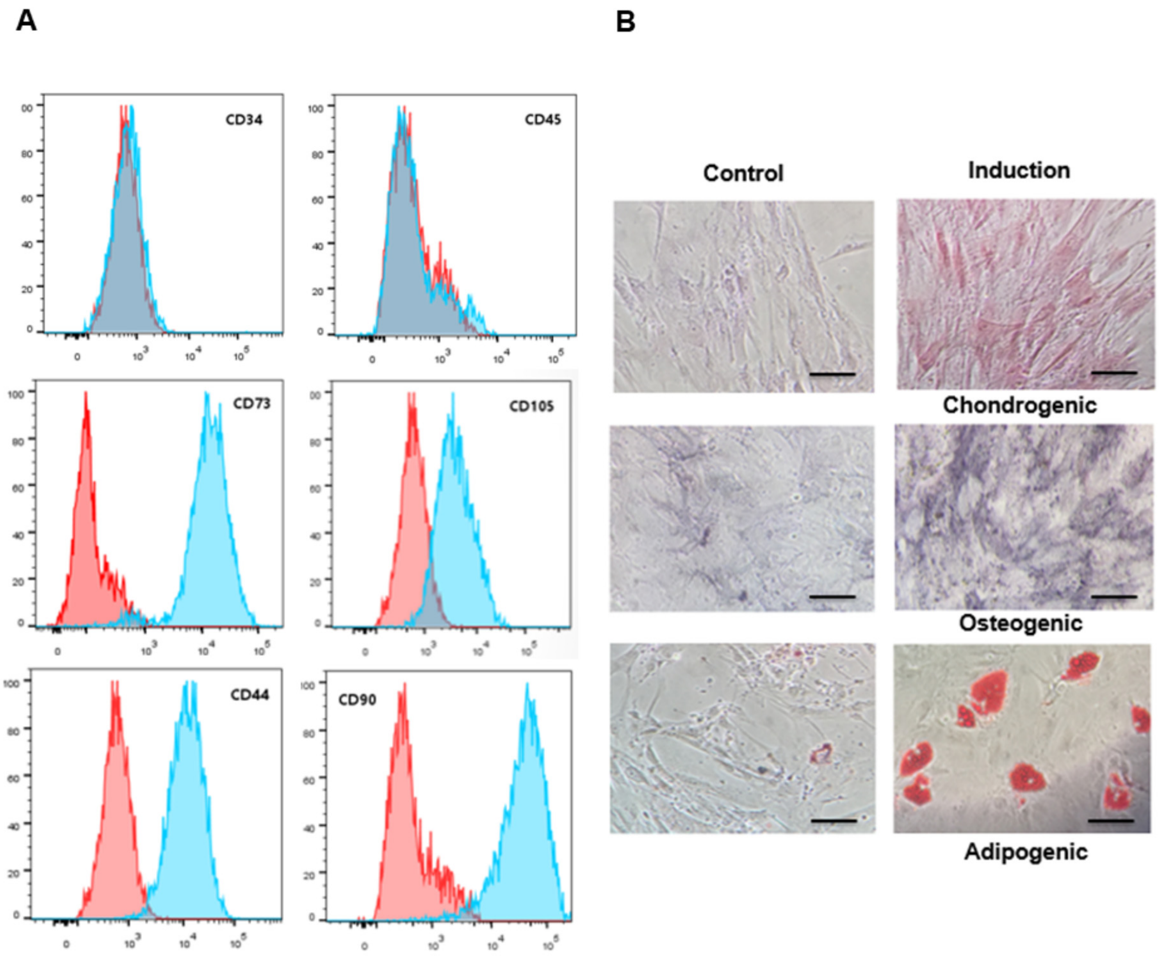

Supplementary Figure S1. Characterization of BM-MSC isolated from healthy orthopedic pediatric patients. (**A**) Isolated and in vitro cultured BM-MSC were characterized by flow cytometry for the cell surface expression of CD105, CD90, CD44, and CD73, and the lack of expression of CD34 and CD45 (blue lines histograms). Unlabeled BM-MSC were used as controls (red lines histograms). (**B**) BM-MSC were also characterized by their ability to differentiate into mesenchymal lineages after specific induction into osteogenic, chondrogenic and adipogenic lineages. After 14-21 days, cells were stained using NBT/BCIP (osteogenic), Safranin O (chondrogenic) and Oil Red (adipogenic) and were microphotographed (20x magnification, 50 μm scale bars) for analysis.

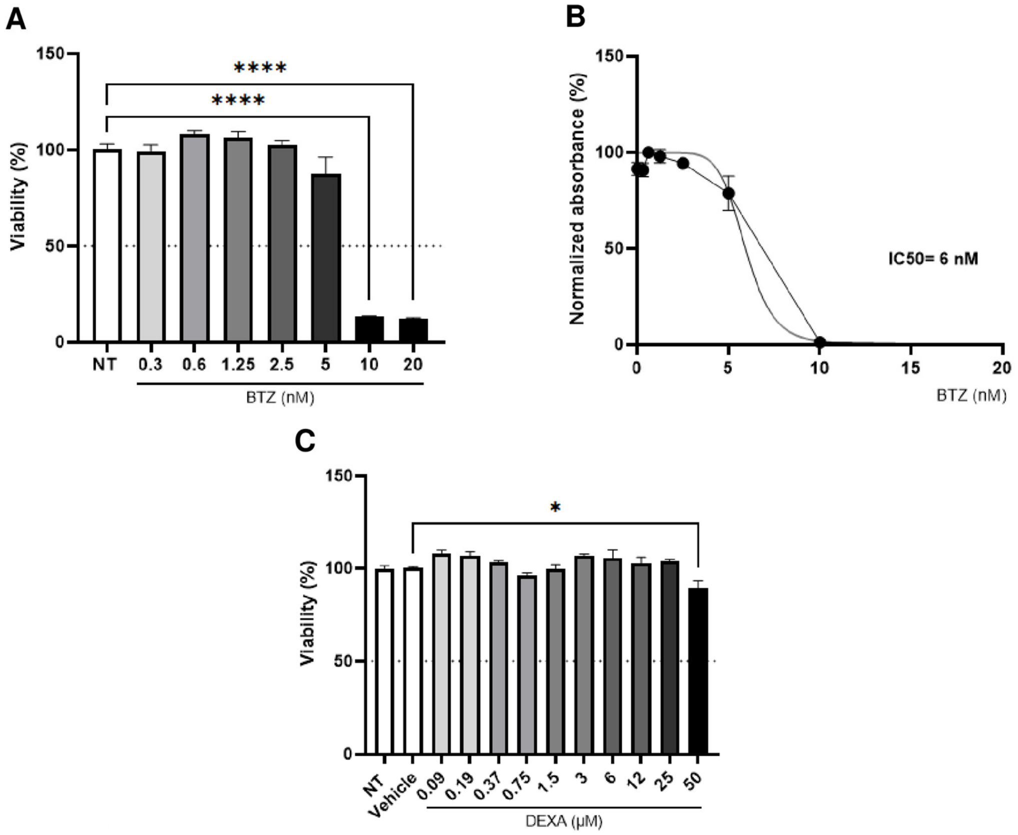

Supplementary Figure S2. H929 cells viability is affected by BTZ treatment but not by DEXA. H929 cells were treated with different concentrations of BTZ **(A)** or DEXA **(C)** for 72 h or with the vehicle (0.4% DMSO) as a control, and cell viability was measured by the MTT assay. **(B)** The IC_{50} was determined from the results obtained in A. Data are expressed as the mean ± SEM of three replicates. Statistical analysis was performed using one-way ANOVA test followed by the Kruskal-Wallis test.*p*-values * < 0.05, **** < 0.0001.

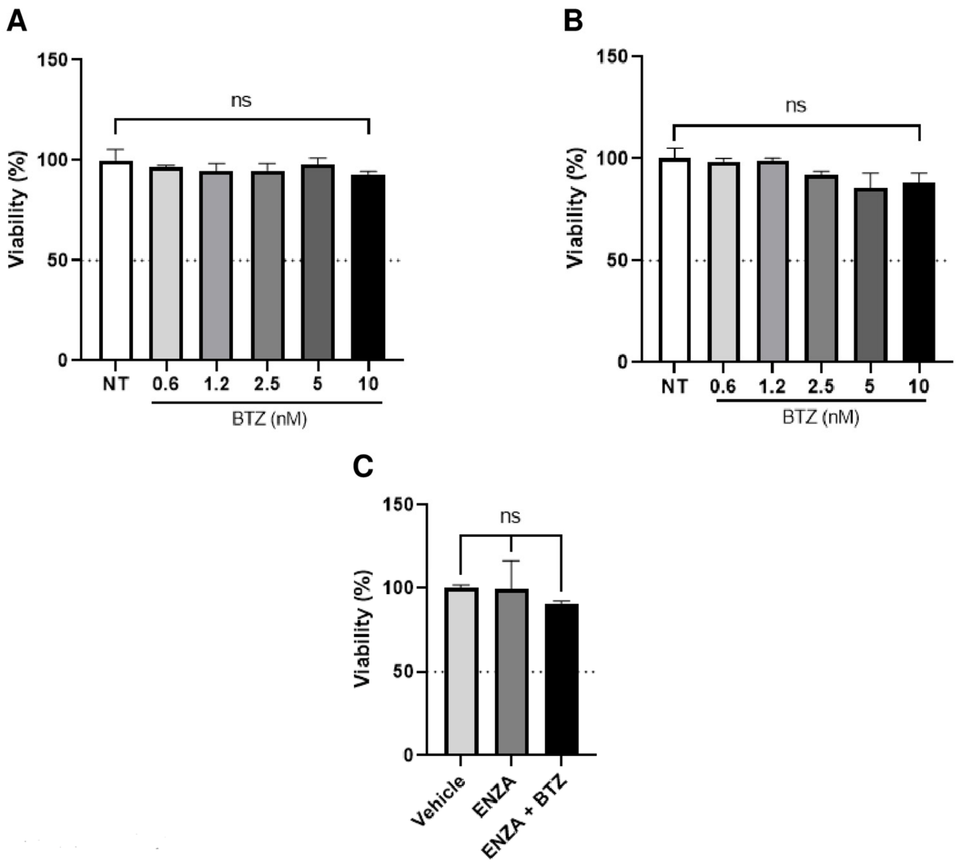

Supplementary Figure S3. BM-MSC viability is not affected by treatments with BTZ and/or ENZA. BM-MSC were treated with BTZ at different concentrations for 24 h **(A)** or 48 h **(B)** and then cell viability was determined by the MTT assay. **(C)** BM-MSC were treated with 20 μM ENZA for 2 h and subsequently treated or not with 20 nM BTZ for 72 h. Treatment with the vehicle (0.4% DMSO) was used as control. Data are expressed as the mean ± SEM of three replicates. Statistical analysis was performed using one-way ANOVA test followed by the Kruskal-Wallis test. NS = not significant.

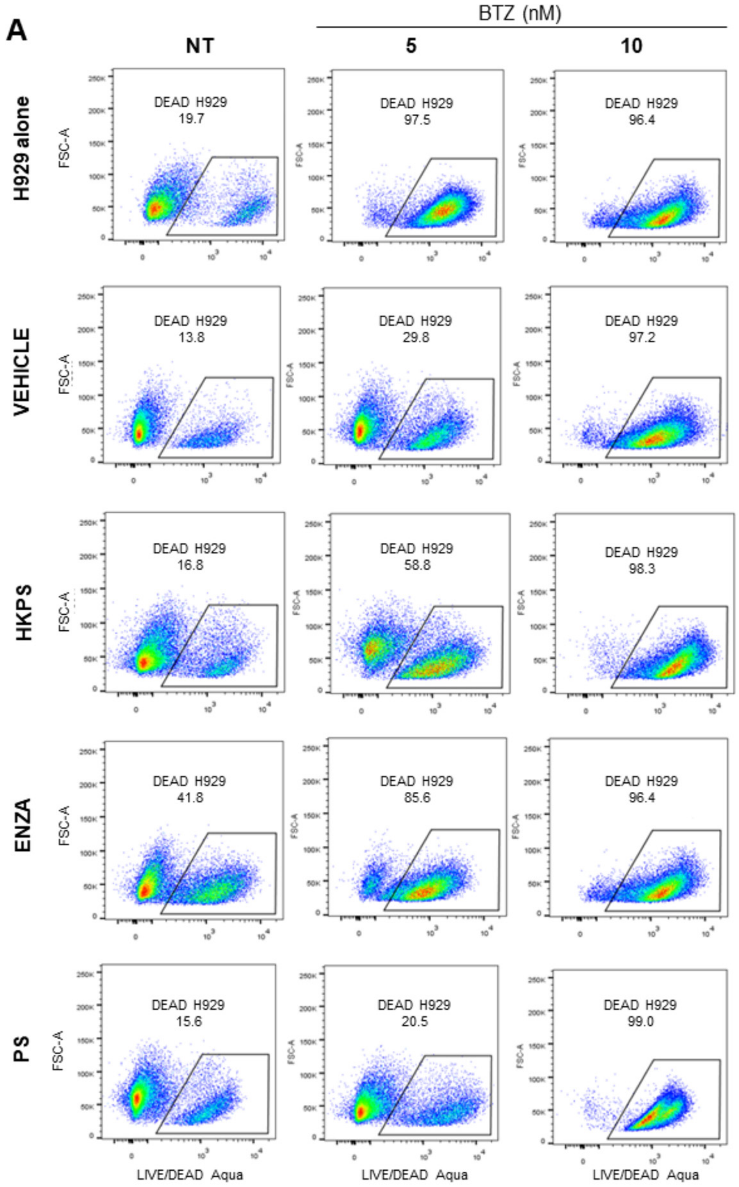

Supplementary Figure S4. Dot blots showing H929 cells sensitivity to BTZ after BM-MSC PKC inhibition. BM-MSC were treated or not with PKC inhibitors (HKPS or ENZA) or the controls the PS peptide or the vehicle (0.4% DMSO) before adding H929 cells, and then co-cultures were treated with BTZ for 72 h at the indicated concentrations. H929 cell viability was assessed by flow cytometry using the LIVE/DEAD Aqua staining. H929 cells alone were used also as a control. A representative experiment is shown. The gate was established for CD73 negative cells from the co-cultures, and the percentage of dead cells was determined as the LIVE/DEAD Aqua staining positive cell population (boxed cells).

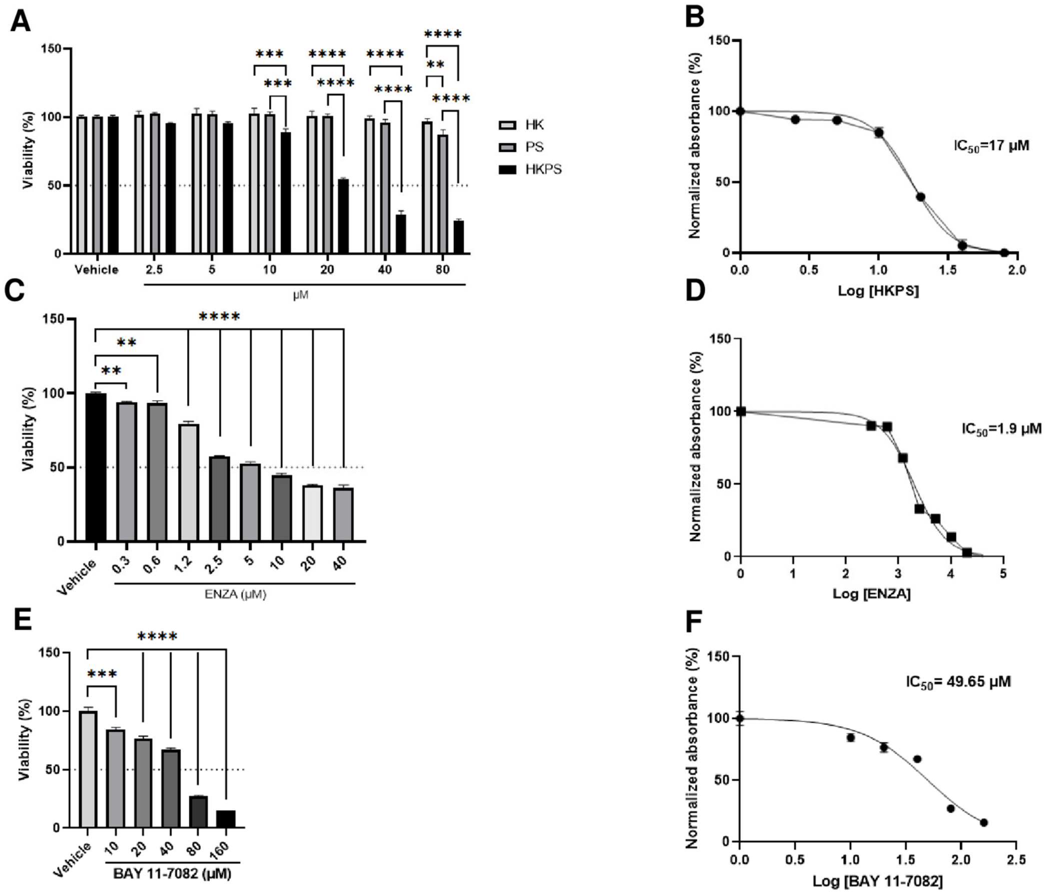

Supplementary Figure S5. H929 cells viability is affected by treatment with HKPS, ENZA or BAY11-0782. H929 cells were treated for 2 h with(A) HKPS, for 4 h with (C) ENZA, or (E) BAY 11-0782. (B, D, F) Results obtained in A, C and E were used to calculate the IC_{50}. Control peptides (HK or PS) or vehicle (NT in A; 0.4% DMSO in C; and 0.02% DMSO in E) were used as controls. Cell viability was determined by the MTT assay. Data are expressed as the mean ± SEM of triplicate cultures. Statistical analysis was performed using one and two-way ANOVA followed by the Dunnett's post-hoc test. *p*-values ** < 0.01, *** < 0.001, **** < 0.0001.

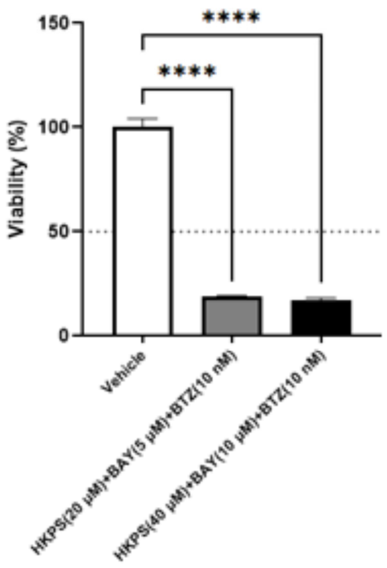

Supplementary Figure S6. Treatment of BM-MSC simultaneously with the inhibitors HKPS and BAY11-7082 followed by BTZ severely affect their viability. BM-MSC alone were simultaneously treated with HKPS (20 or 40 μM), BAY11-7082 (5 or 10 μM), and BTZ (10 nM) for 24 h; BM-MSC treated with vehicle (0.02% DMSO) were used as a control. Data are expressed as the mean ± SEM of three replicates. Statistical analysis was performed using one-way ANOVA followed by the Kruskal-Wallis test. *p*-values **** < 0.0001.
