# Supplementary figures and images for: Simultaneously Targeting Two Coupled Signalling Molecules in the Mesenchymal Stem Cell Support Efficiently Sensitises the Multiple Myeloma Cell Line H929 to Bortezomib
Source: Int J Mol Sci. 2023 May 2;24(9):8157. doi: 10.3390/ijms24098157 (PMC10178910; doi:10.3390/ijms24098157)

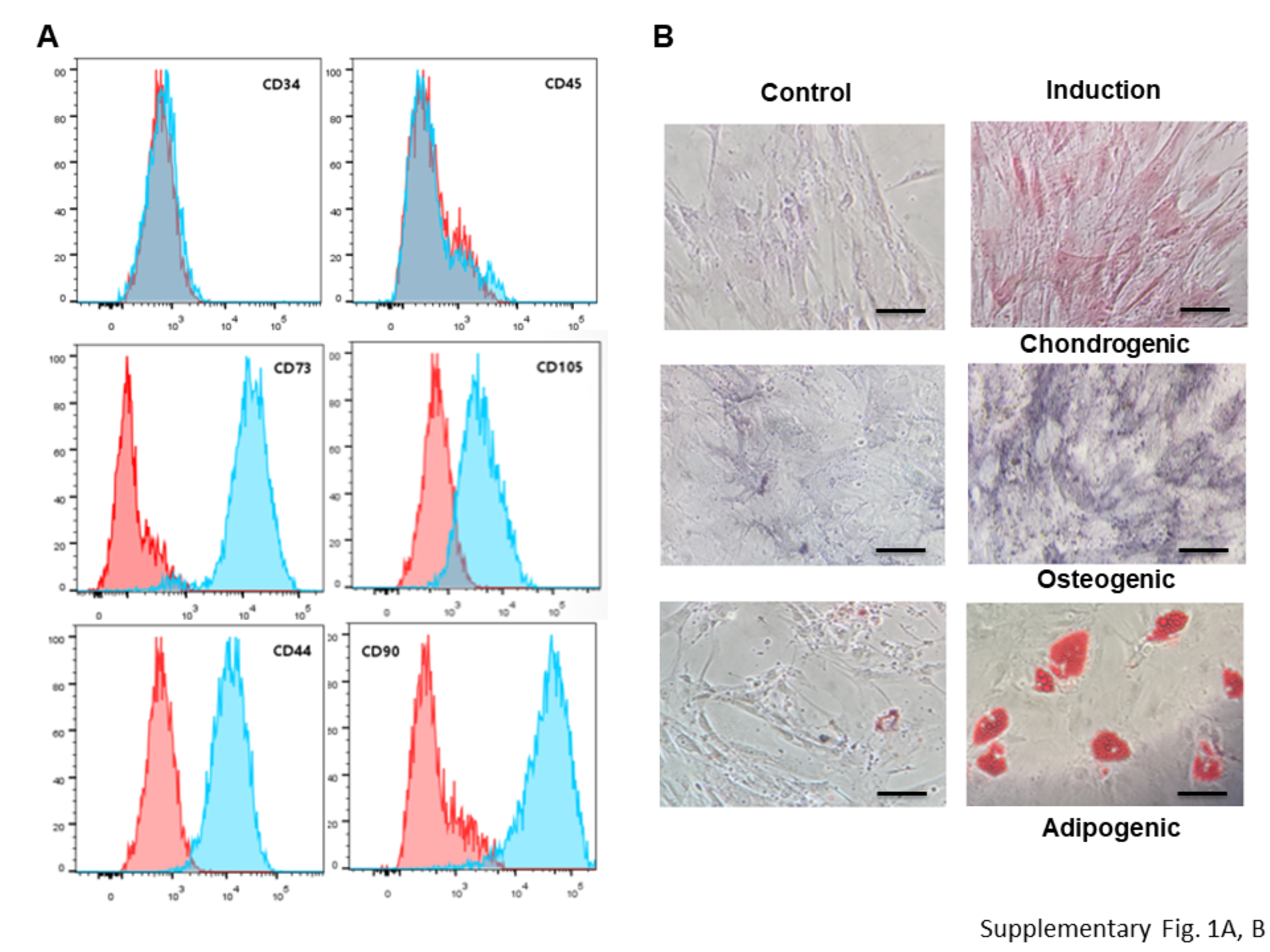

Supplement: Supplementary file 1 [file ijms-24-08157-s001.zip › Figure S1.tif]

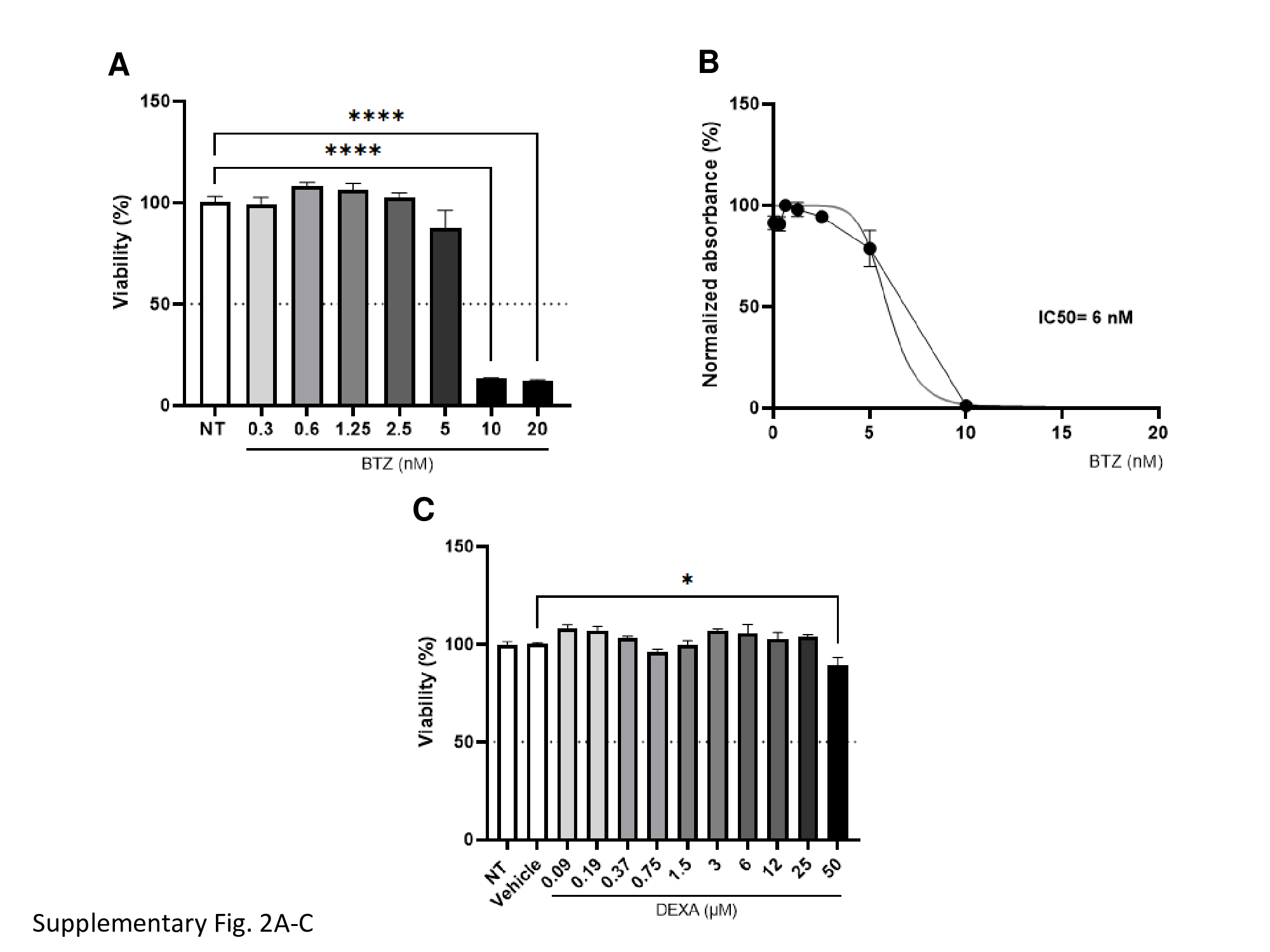

Supplement: Supplementary file 1 [file ijms-24-08157-s001.zip › Figure S2.tif]

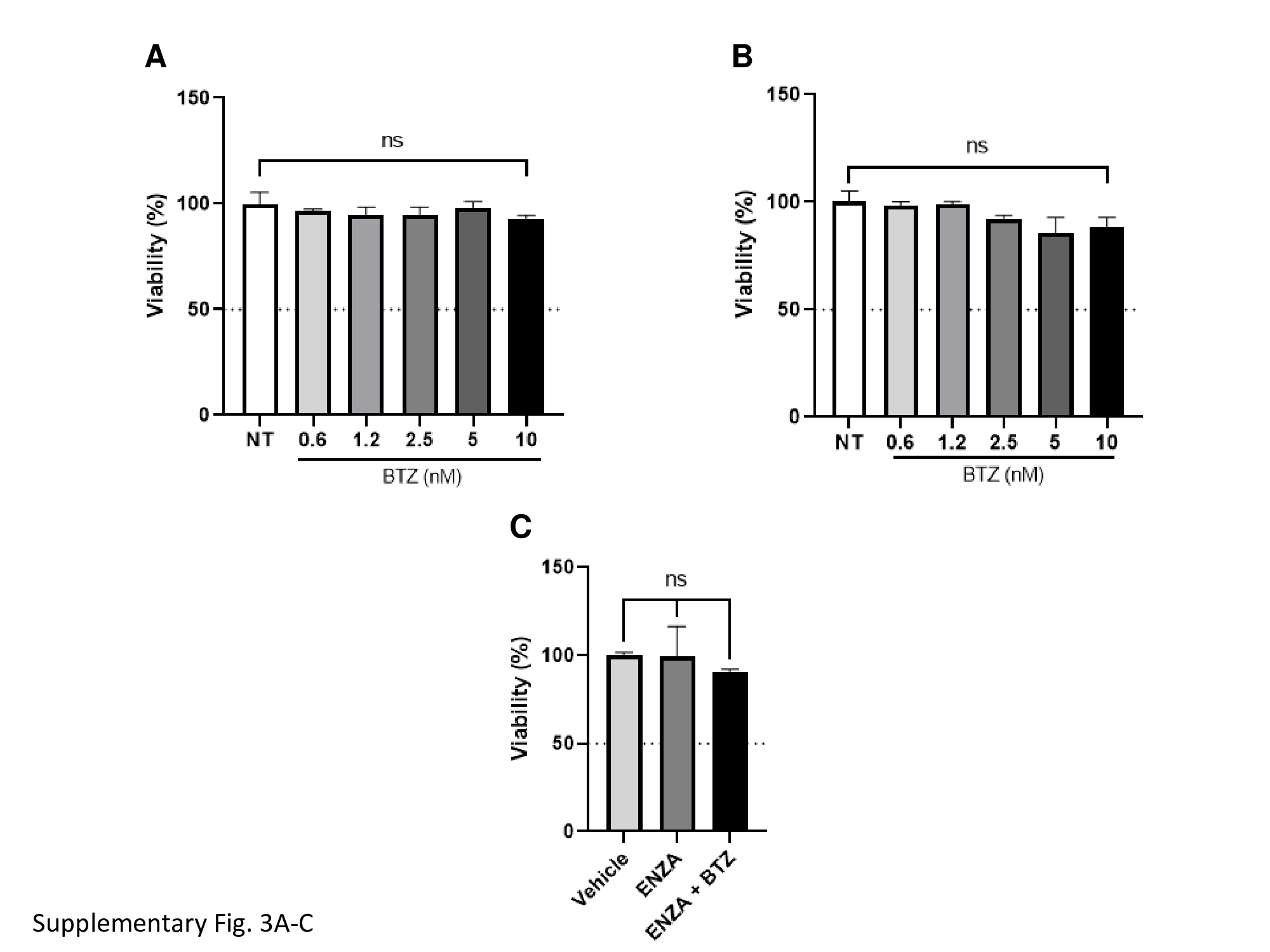

Supplement: Supplementary file 1 [file ijms-24-08157-s001.zip › Figure S3.tif]

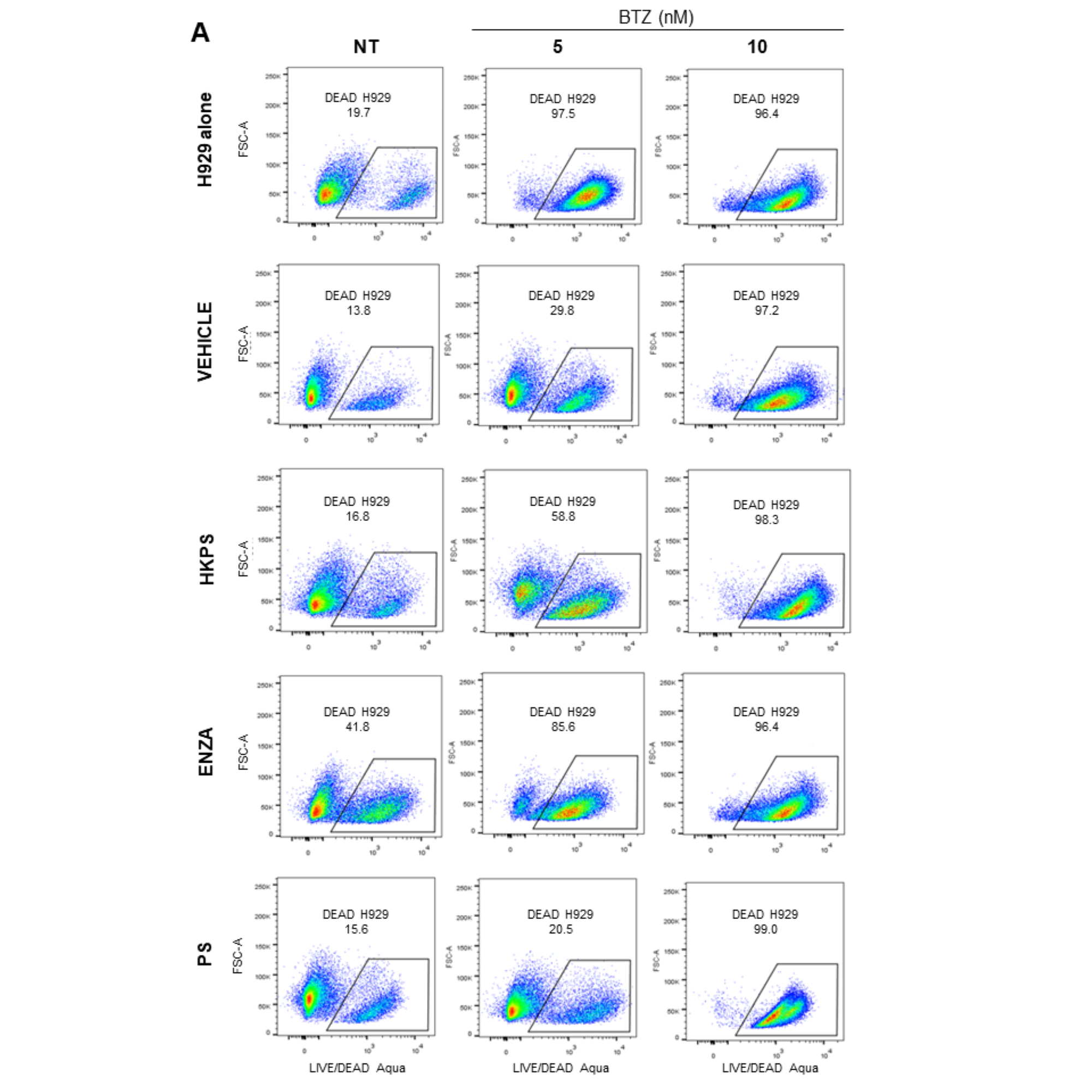

Supplement: Supplementary file 1 [file ijms-24-08157-s001.zip › Figure S4.tif]

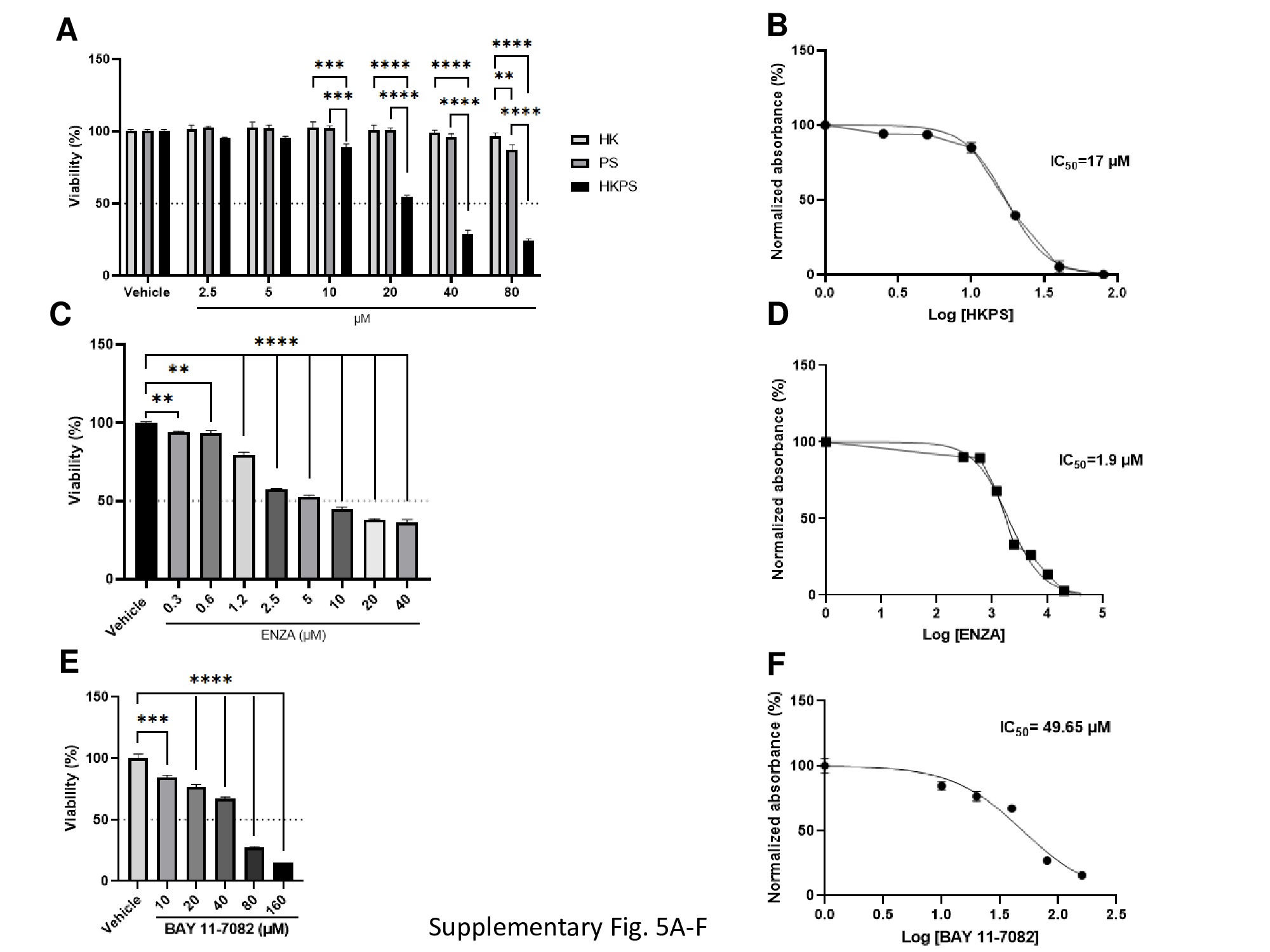

Supplement: Supplementary file 1 [file ijms-24-08157-s001.zip › Figure S5.tif]

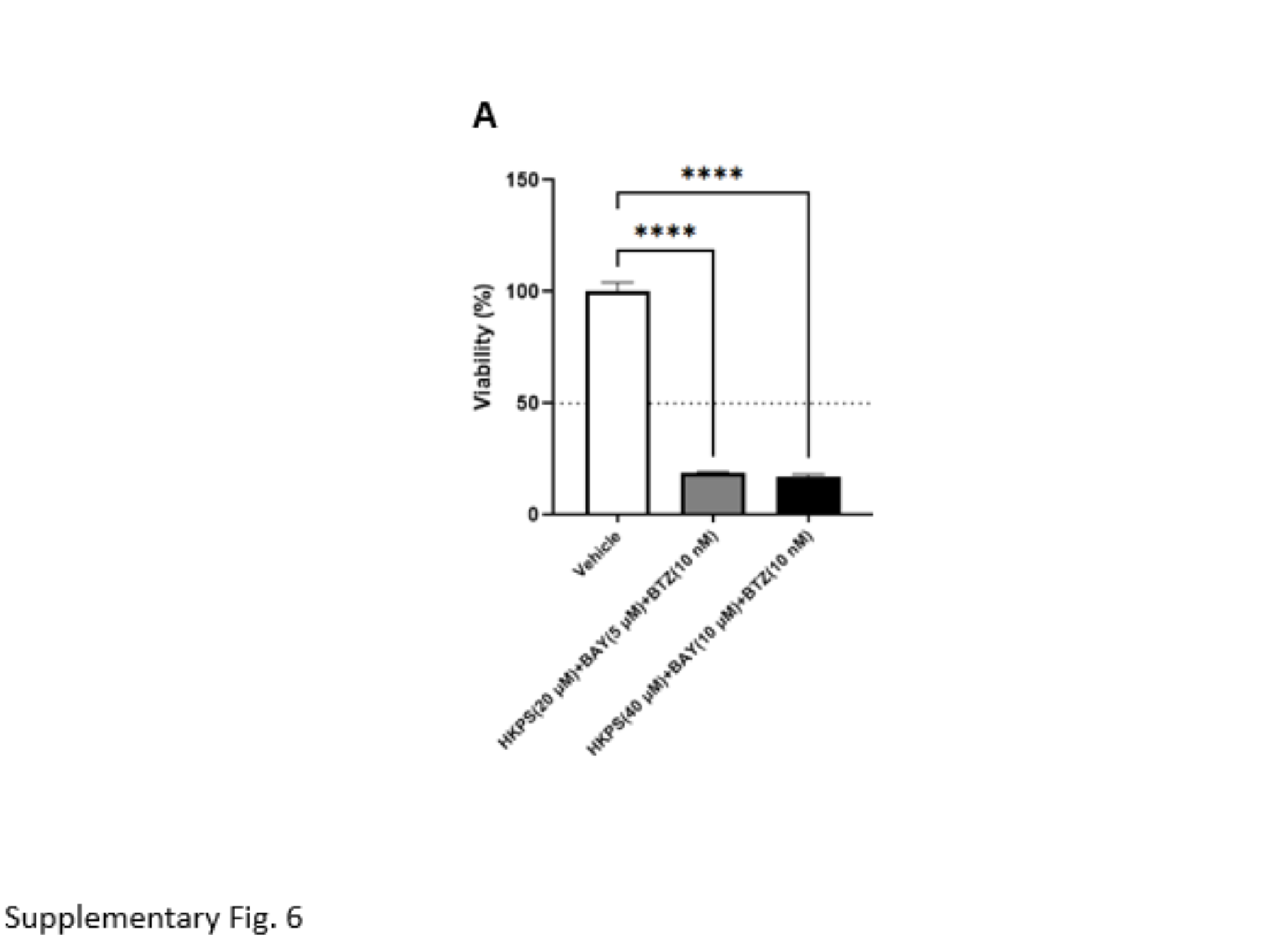

Supplement: Supplementary file 1 [file ijms-24-08157-s001.zip › Figure S6.tiff]
